# What are the methodological designs of nihr funded clinical trials? Going beyond routine data collection

**DOI:** 10.1186/1745-6215-16-S2-P43

**Published:** 2015-11-16

**Authors:** Amanda Young, Helen Buxton, Liz Tremain, Tom Kenny

**Affiliations:** 1 National Institute for Health Research, Evaluation, Trials and Studies Coordinating Centre, Southampton, UK

## Background

In 2015, a report was published in the Health Technology Assessment which piloted the use of extracting metadata on the design, conduct, results and costs of clinical trials funded by the Health Technology Assessment programme. A recommendation was to explore the feasibility of extracting data on the active NIHR portfolio of clinical trials.

## Aims and objectives

To develop and pilot a method to capture data on the design and methodology of all active NIHR funded clinical trials.

## Method

We will review the NIHR portfolio of clinical trials that have an active status (from pre-contracting status to submission of final report) from data extracted in November 2014. The first part of the pilot will extract data on the design and methodological features of a clinical trial, using the existing data fields from the metadata database published in the 2015 HTA report.

## Results

The key findings will include an overview of the type of clinical trials funded by NIHR and whether there are differences between the NIHR Programmes. Particular attention will be paid to key methodological elements such as the design framework, number of arms and primary outcomes and the type of interventions.

## Conclusions

Examining the methodological design of NIHR clinical trials is important to maximise the added value of metadata approaches beyond routine data collection and for increasing awareness of the type of trials funded by NIHR.

